# Time-dependent LXR/RXR pathway modulation characterizes capillary remodeling in inflammatory corneal neovascularization

**DOI:** 10.1007/s10456-018-9604-y

**Published:** 2018-02-14

**Authors:** Anthony Mukwaya, Anton Lennikov, Maria Xeroudaki, Pierfrancesco Mirabelli, Mieszko Lachota, Lasse Jensen, Beatrice Peebo, Neil Lagali

**Affiliations:** 1 Department of Ophthalmology, Faculty of Health Sciences, Institute for Clinical and Experimental Medicine, Linkoping University, 58183 Linköping, Sweden; 2 Department of Immunology, Medical University of Warsaw, Warsaw, Poland; 3 Division of Cardiovascular Medicine, Department of Medical and Health Sciences, Linköping University, Linköping, Sweden

**Keywords:** Cornea neovascularization, Inflammation, Angiogenesis, Remodeling

## Abstract

Inflammation in the normally immune-privileged cornea can initiate a pathologic angiogenic response causing vision-threatening corneal neovascularization. Inflammatory pathways, however, are numerous, complex and are activated in a time-dependent manner. Effective resolution of inflammation and associated angiogenesis in the cornea requires knowledge of these pathways and their time dependence, which has, to date, remained largely unexplored. Here, using a model of endogenous resolution of inflammation-induced corneal angiogenesis, we investigate the time dependence of inflammatory genes in effecting capillary regression and the return of corneal transparency. Endogenous capillary regression was characterized by a progressive thinning and remodeling of angiogenic capillaries and inflammatory cell retreat in vivo in the rat cornea. By whole-genome longitudinal microarray analysis, early suppression of VEGF ligand-receptor signaling and inflammatory pathways preceded an unexpected later-phase preferential activation of LXR/RXR, PPARα/RXRα and STAT3 canonical pathways, with a concurrent attenuation of LPS/IL-1 inhibition of RXR function and Wnt/β-catenin signaling pathways. Potent downstream inflammatory cytokines such as Cxcl5, IL-1β, IL-6 and Ccl2 were concomitantly downregulated during the remodeling phase. Upstream regulators of the inflammatory pathways included Socs3, Sparc and ApoE. A complex and coordinated time-dependent interplay between pro- and anti-inflammatory signaling pathways highlights a potential anti-inflammatory role of LXR/RXR, PPARα/RXRα and STAT3 signaling pathways in resolving inflammatory corneal angiogenesis.

**Electronic supplementary material:**

The online version of this article (10.1007/s10456-018-9604-y) contains supplementary material, which is available to authorized users.

## Introduction

Pathologic inflammation in the normally immune-privileged cornea can lead to vision loss and blindness and can result from surgical procedures such as corneal transplantation, after infection or following traumatic injury (physical or chemical). Early in the inflammatory response, the local injury induces cellular apoptosis and cytokine signaling facilitating the migration of leukocytes from capillaries and tissue in the surrounding limbal and conjunctival region to the cornea at the affected site [1, 2]. The inflammatory cells promote further cytokine production building a cytokine concentration gradient leading to subsequent dilation of limbal capillaries and angiogenic sprouting [3]. Molecular cross talk between inflammation and angiogenesis mediated partly by cytokines has been described in the eye; for instance, Amaral et al. [4], illustrated the dual pro-angiogenic and pro-inflammatory properties of 7kCh. Other inflammatory factors, for example TNF-α [5], IL-1 [6], Il-8 [7] and IL-1β [8], are reported to be important for corneal neovascularization. A neutrophil-mediated proteoglycan/CXCL1 complex has been shown to disrupt the chemokine gradient to resolve corneal inflammation [9]. Also, TNF-α-stimulated protein 6 (TSG-6), a protein with anti-inflammatory activity, was shown to reduce corneal neovascularization by inhibiting neutrophil infiltration into the rat cornea in an alkali burn model [10]. In other tissues, interleukins IL-6 and IL-17 have been shown to modulate the expression of VEGF [11, 12], while chemokines CXCL8 and CXCL12 have been implicated in both inflammation and angiogenesis [13].

Resolving inflammation can be crucial for managing corneal neovascularization; however, the various activated inflammatory pathways and their temporal importance are still poorly understood. Corneal inflammation can naturally subside over time [14], and capillary regression is reported to be associated with a build-up of macrophages [15] of the M2 phenotype [16]. Pathways such as NF-κB, PI3 K and cAMP are involved in the apoptotic resolution of inflammation [17], but this has only been shown in a defined cell type. Despite the identification of a number of important inflammatory cytokines and factors involved in pathologic corneal inflammation and angiogenesis, such factors are part of broader inflammatory pathways acting in a coordinated manner. Clinically, broad-acting steroids are widely used to suppress inflammation; however, these target many inflammatory and physiological pathways simultaneously and indiscriminately, leading to a risk of adverse side effects including glaucoma and cataracts. A deeper investigation of key inflammatory pathways involved in inflammation and the resulting angiogenic response could therefore pave the way for improved therapies targeted against specific activated inflammatory pathways, or alternatively, promoting pathways associated with the resolution of inflammation and capillary remodeling.

We previously demonstrated that suppression of inflammatory and angiogenic genes is characteristic of induced capillary remodeling in the early resolution phase [16]. In this study, we hypothesized that not just an initiation of inflammatory pathway suppression is important for vessel regression, but a sustained suppression of inflammation pathways and a concurrent activation of other vascular remodeling pathways characterize the regression and remodeling process over time. Here, we investigate the pathways evoked during the time-dependent modulation of inflammation occurring during active corneal angiogenesis and during its subsequent endogenous resolution. Using whole transcriptome microarrays for the rat, gene expression profiles of neovascularized corneas under sustained inflammatory stimulation (by intrastromal suture placement) were compared to corneas where inflammation was stimulated to rapidly resolve (by the removal of the suture stimulus). The differential expression profiles in sustained inflammation and angiogenic sprouting versus dampened inflammation and capillary regression/remodeling were analyzed at both the gene and pathway level in a time-dependent manner, to gain insights into the inflammatory pathway dynamics.

## Results

### Hierarchical cluster analysis reveals time-dependent differences in inflammatory angiogenic sprouting and capillary remodeling at the whole transcriptome level

**Fig. 1:**
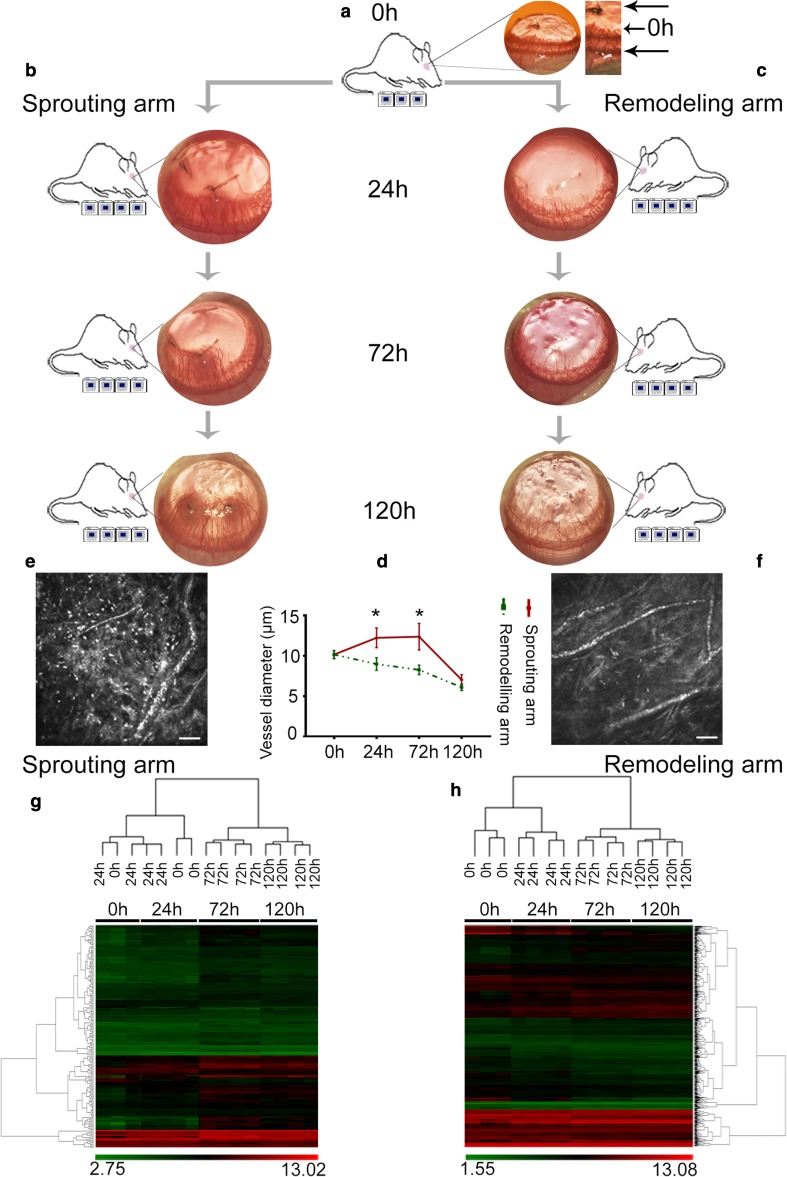
Phenotypic analysis of the neovascularization response and hierarchal cluster analysis of samples in the sprouting and remodeling arms. In the experimental layout used in the study, **a** is the 0 h time point when angiogenic sprouts extended halfway to the sutures as indicated in the zoomed-in image. **b** The sprouting arm and **c** is the remodeling arm, and both were defined from 0 h onward. In each arm, the 24-, 72- and 120-h time points are relative to the 0-h time point, and the digital images at each of these time points show the neovascularization response with neovessels originating from the limbus and sprouting toward the center of the cornea. Indicated below each schematic representation of the rat is the number of microarray chips used for analysis at the given time point. For 0 and 24 h, we retrieved microarray raw data from our previous NCBI submission [85]. **d** Capillary diameter over time, measured from the IVCM image sequences in **e** and **f**. **e** and **f** are representative IVCM images taken at 72 h showing perfused capillaries and the cellular infiltrate in the vascularized cornea. Inflammatory cells infiltrate the cornea originating from the limbus, and this starts a few hours following stimulation by suture placement. Scale bar in **e** and **f** = 50 µm, *n* = 4, and the error bars in **d** represent SEM. **g, h** are the hierarchical clusters from the analysis of the samples using the CHP files generated from normalizing the microarray raw CEL files, and below each hierarchical cluster are the corresponding heat maps showing linear signal intensity values. The signal intensity values were sorted on ANOVA *p* < 0.05 and FDR < 0.1, to generate the heat maps

A model of suture-induced inflammatory angiogenesis in the rat cornea was used to investigate inflammation-mediated remodeling of a new angiogenic capillary plexus in the cornea over time. Two nylon sutures placed intrastromally induced an early inflammatory response followed by sprouting from pre-existing capillaries at the limbus in a direction toward the sutures. When new capillary sprouts reached halfway to the suture (designated as time point 0 h), separate groups of rats were divided into remodeling and sprouting arms. In the remodeling arm, sutures were removed at 0 h to induce subsequent capillary regression, while in the sprouting arm sutures were retained to provide a sustained stimulus for inflammatory angiogenesis (Fig. 1a–c) [16]. From digital images used to monitor the neovascularization response, edema and inflammation were visibly dampened in the remodeling arm relative to the sprouting arm during the 24–120-h period (Fig. 1b, c). Invading inflammatory cells persisted in the corneal stroma in the sprouting arm but not in the remodeling arm as indicated by IVCM examination (Fig. 1c, f and supplementary Fig. 1). In addition, macrophages were the dominant inflammatory cell type present at 72 and 120 h in the remodeling arm (Supplementary Fig. 1b). Also using in vivo confocal microscopy to monitor capillary perfusion and cellular infiltration in the cornea, it was observed that capillaries constricted with time in both sprouting and remodeling arms (ANOVA *p* = 0.0002 and *p* < 0.0001, respectively). However, the constriction was greater in the remodeling arm at 24 and 72 h (*p* = 0.007 and *p* = 0.006, respectively) (Fig. 1d and supplementary Fig. 1). Hierarchical cluster analysis of the whole transcriptome microarray data across individual rats revealed clustering at given time points. In the sprouting arm, an overlap occurred between 0 and 24 h; however, a distinctive clustering pattern followed from 24 to 120 h (Fig. 1g). In the remodeling arm, samples clustered separately according to time point (Fig. 1h). The clustering pattern observed in both the sprouting and remodeling arms is indicative of strong time-dependent transcriptomic responses.

Following the observed phenotype, the whole transcriptome data were then analyzed to gain insights into the associated differentially expressed genes (DEG) and their expression profile with time in the sprouting and remodeling arms.

### Time course analysis of DEG indicates a greater number and degree of downregulated genes during remodeling

**Fig. 2:**
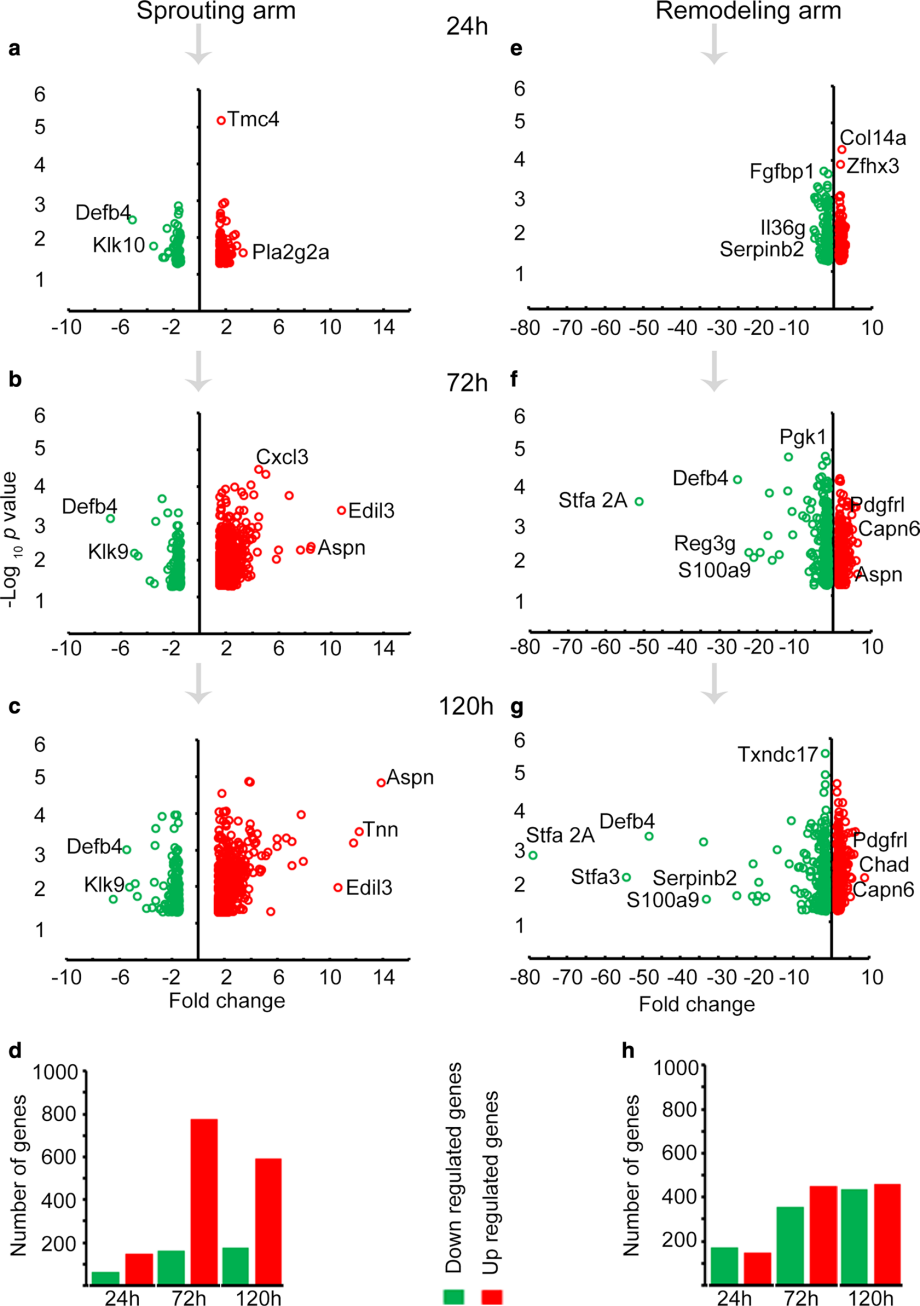
DEG across time points in sprouting and remodeling arms. **a–c** are volcano plots for the DEG at 24-, 72- and 120-h time points in the sprouting arm, while **e–g** are the corresponding plots in the remodeling arm. For both groups of volcano plots, the red small circles represent upregulated genes, while green small circles represent downregulated genes, both relative to the 0-h time point. Genes labeled in each volcano plot exemplify high fold change or *p* value, or both, over time. In **d**, **h** is the number of up- and downregulated genes among the DEG at each time point and across time points in the sprouting and remodeling arms, respectively. The upregulated genes are represented by the red-colored bars, while the downregulated genes are represented by the green-colored bars

With focus on the whole transcriptome data, DEG were identified across time points in the two arms. To do this, the 0-h time point was assigned as a baseline to normalize the gene expression data from the other time points. Genes were then filtered using fold change ≥ 1.5 or ≤ −1.5 and *p* value < 0.05. Based on these parameters, we obtained 208, 936 and 763 DEG at 24-, 72- and 120-h time points, respectively, in the sprouting arm (Fig. 2a–c). In the remodeling arm, 315, 806 and 893 DEG were identified at 24-, 72- and 120-h time points, respectively (Fig. 2e–g). To further assess the differential expression pattern between sprouting and remodeling arms, Venn diagram analysis was performed [18] to depict any overlap of genes between the two arms. It was noted that with time the percentage of genes common to both arms increased (Supplementary Fig. 2). In terms of fold change expression, downregulation of genes was much stronger in the remodeling arm compared to the sprouting arm. For instance, at 72 h beta-defensin (Defb4) was suppressed sevenfold in the sprouting arm and 25-fold in the remodeling arm (Fig. 2b, f). In addition, the downregulated genes within the remodeling arm were further suppressed with time. For instance, a gene, similar to stefin A2 (Stfa 2A), was suppressed 50-fold at 72 h and 80-fold at 120 h (Fig. 2f, g). Moreover, the number of downregulated genes was greater at all time points in the remodeling arm than in the sprouting arm (Fig. 2d, h).

To investigate the biological significance of the DEG described above, pathway enrichment analysis was performed using IPA software. A core analysis was performed using the DEG and then analyzed for canonical pathway enrichment in both arms at all time points.

### Differential pathway analysis reveals time-dependent inflammatory pathway inhibition and remodeling pathway activation

**Fig. 3:**
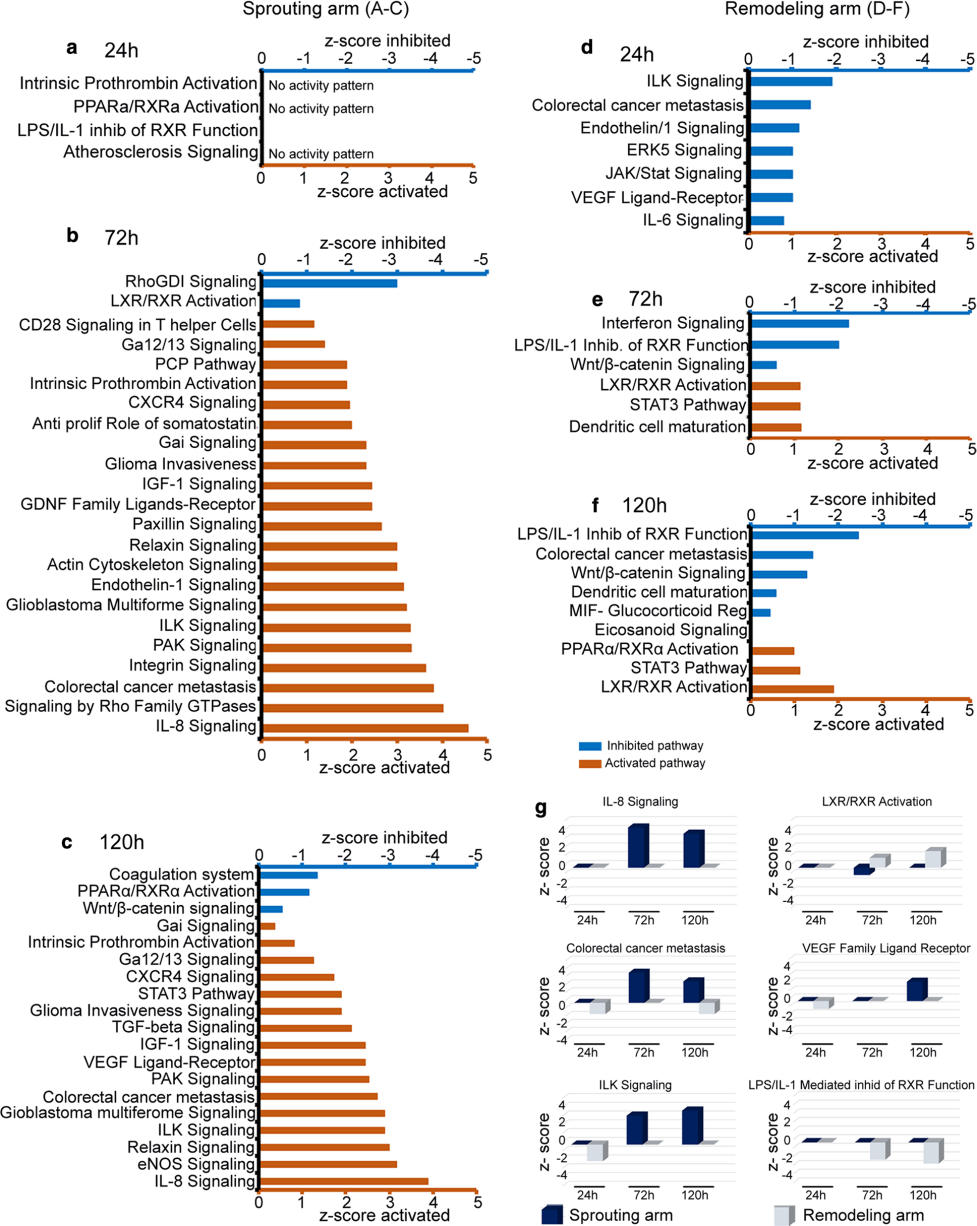
Modulation of canonical pathways in the sprouting and remodeling arms with time. The DEG generated at the different time points were used for pathway enrichment analysis, and all pathways significantly enriched with a significance *p* value < 0.05 are shown in **a**–**f**, irrespective of their *z*-score value. **a–c** show pathways enriched at 24-, 72- and 120-h time points, respectively, in the sprouting arm. **d–f** show pathways enriched at 24-, 72- and 120-h time points, respectively, in the remodeling arm. In **a–f**, the blue bars represent inhibited pathways, while the orange bars represent activated pathways based on the *z*-score. Pathways significantly enriched, but with no activity pattern available are indicated with the extra statement ‘no activity pattern available.’ Pathway ‘LPS/IL-1 inhibition of RXR Function’ in **a** had *z*-score = 0, i.e., neither active nor inhibited. For **a–f**, on the *x*-axis the negative *z*-score values represent strength of inhibition, while positive *z*-score values represent strength of activation. On the *y*-axis are the names of the enriched pathways. **g** is a closer comparison of selected pathways between the sprouting and remodeling arms at 24-, 72- and 120-h time points. In **g**, dark blue bars represent the sprouting arm, while gray bars represent the remodeling arm

The enriched pathways were initially filtered for a significant overlap between DEG and the QIAGEN Knowledge Base, with *p* value < 0.05 considered significant, and the pathways that met this criterion were further analyzed. The time-dependent pathway analysis across arms indicated two phases of pathway enrichment in the remodeling arm (Fig. 3d–f). In the first phase, several key inflammatory and angiogenic pathways were inhibited early at 24 h, including ILK signaling, IL-6 signaling, endothelin-1 signaling, VEGF family receptor ligand signaling, Jak/stat and ERK5 signaling (Fig. 3d).

In a second, later phase at 72 h, LXR/RXR and STAT3 pathways were activated in the remodeling arm, along with the inhibition of the pathway ‘LPS/IL-1 inhibition of RXR function’ (Fig. 3e). LXR/RXR pathway was conversely inhibited in the sprouting arm at the same time point (Fig. 3b). The activation/inhibition pattern of these three pathways persisted at 120 h in the remodeling arm (Fig. 3f). In addition, a host of inflammatory pathways activated at 72 h in the sprouting arm were no longer active in the remodeling arm (Fig. 3b, e). Furthermore, the PPARα/RXRα activation pathway was differentially modulated between the sprouting (inhibited) and remodeling (activated) arms (Fig. 3c, f). The analysis also revealed other pathways of potential interest in the resolution of inflammation and remodeling, such as IL-8 signaling, colorectal cancer metastasis and Wnt/beta-catenin signaling.

An extended canonical pathway enrichment analysis in the sprouting and remodeling arms is found in supplementary Fig. 3, which illustrates the relationship between the significance of overlap between the DEG and QIAGEN Knowledge Base (presented as a significance score; negative log of *p* value calculated using Fisher’s exact test) and activity *z*-score.

In the remodeling arm, activated pathways LXR/RXR activation and STAT3, and inhibited pathways Wntβ catenin signaling and LPS/IL-1 inhibition of RXR function, exhibited persistence in activation/inhibition status from 72 to 120 h (Fig. 3e, f). With a focus on the remodeling arm, we next identified and evaluated the time dependence of genes involved in these pathways.

### Genes in the enriched pathways within the remodeling arm exhibit time-dependent expression patterns

**Fig. 4:**
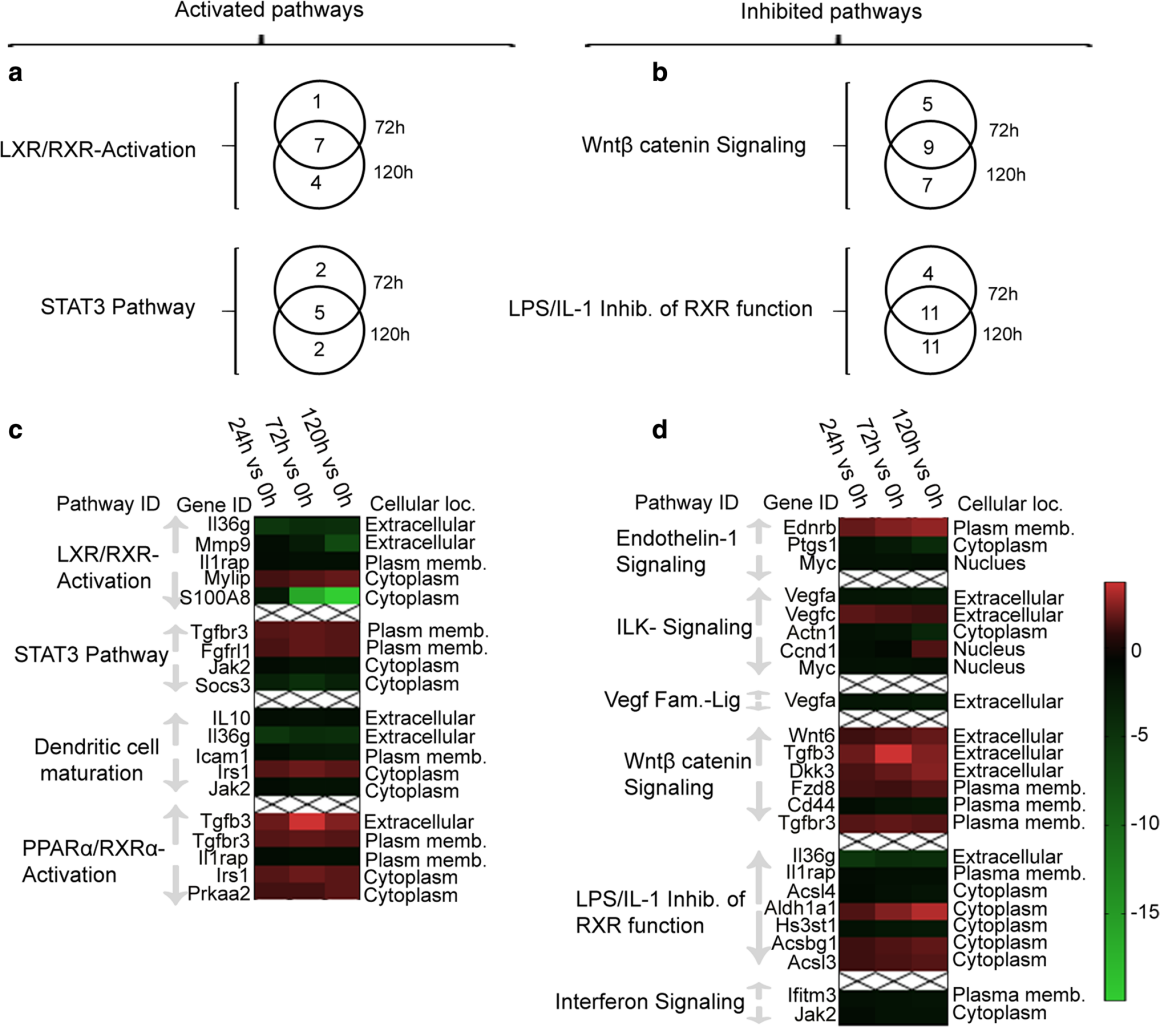
Time dependence of DEG expression within enriched pathways of interest in the remodeling arm. Only pathways enriched at both 72 and 120 h in the remodeling arm were considered and are shown in a and b. **a** is a Venn diagram representation of DEG involved in activated pathways LXR/RXR activation and STAT3, at 72- and 120-h time points. **b** is a Venn diagram representation of DEG involved in the inhibited pathways Wntβ catenin signaling and LPS/IL-1 inhibition of RXR function, at 72- and 120-h time points. Numbers in the Venn diagram indicate number of DEGs present in the pathway at the given time point and at both time points (overlap region). Pathways of interest were also selected from the remodeling arm irrespective of their time dependence, and the genes within them analyzed. The heat maps in **c** (genes isolated from selected activated pathways) and **d** (genes isolated from selected inhibited pathways) illustrate the fold change of selected genes within these pathways over time. The red color represents upregulated genes, while the green color represents downregulated genes relative to 0 h. Only genes with *p* value < 0.05 in at least two time points relative to the 0-h time point are represented in the heat maps in **c** and **d**

DEG within the canonical pathways enriched during the later remodeling phase as described above, were compared between 72 and 120 h in the remodeling arm. Many of the genes in the activated pathways LXR/RXR activation and STAT3, as well as those in the inhibited pathways Wntβ catenin signaling and LPS/IL-1 inhibition of RXR function, were common to both time points (Fig. 4a, b).

In addition, temporal variation of genes within a selection of pathways enriched in the remodeling arm was assessed from 24 to 120 h relative to 0 h (time of suture removal), with all examined genes having a *p* value < 0.05 between sprouting and remodeling arms (Fig. 4c, d). Pro-inflammatory cytokines such as S100 calcium-binding protein A8 (S100A8) and matrix metalloproteinase-9 (Mmp9) were progressively suppressed over time in the LXR/RXR signaling pathway, while Vegfa and Vegfc were progressively suppressed in ILK and VEGF ligand-receptor signaling pathways.

Given the observed differential modulation profile of liver X receptors (LXRs) with time, and between arms, we looked deeper into the expression pattern of these receptors (LXRα, LXRβ) by immunofluorescence and by Western blot, respectively.

### LXRs are expressed in the cornea in different cell populations

**Fig. 5:**
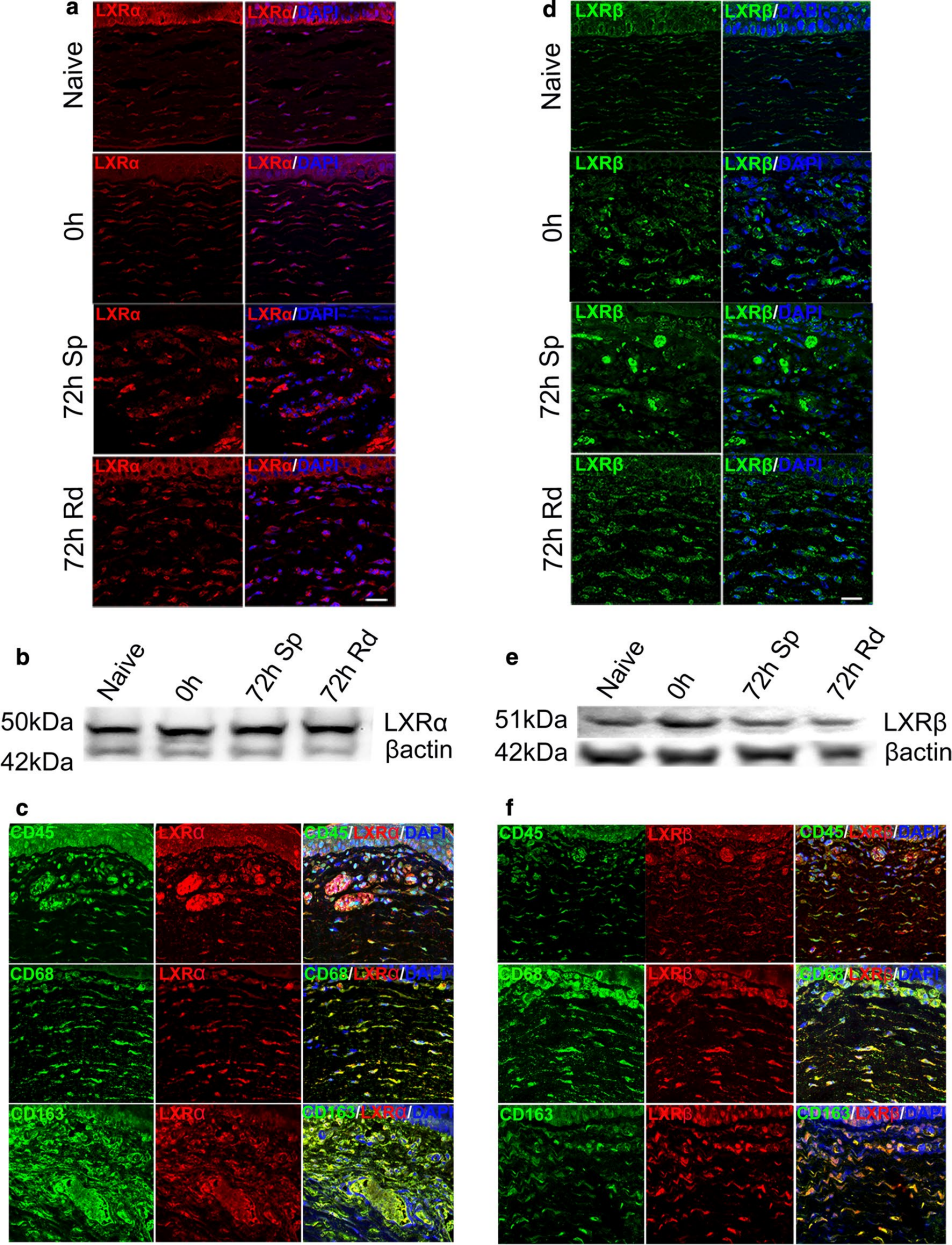
Expression of LXRs in cornea cross sections (by immunofluorescence) and in lysates (by Western blot analysis) in the naïve cornea, at 0 h and at 72 h within the sprouting (Sp) and remodeling (Rd) arms. **a** and **d** are confocal immunofluorescence images showing the localization of LXRα (red) and LXRβ (green), respectively, in cornea tissue sections. **b** and **e** are Western blots for LXRα and LXRβ, respectively, from cornea lysates in the naïve cornea, at 0-h and at 72-h time points. **c** are confocal immunofluorescence images of LXRα co-expression in CD45+, CD68+ and CD163+ cells in cornea cross sections at 72 h in the remodeling arm. The red color represents LXRα, while the green color represents a given cell marker. **f** are confocal immunofluorescence images of LXRβ co-expression in CD45+, CD68+ and CD163+ cells in cornea cross sections at 72 h in the remodeling arm. The red color represents LXRβ, while the green color represents a given cell marker. The scale bar in the each of the merged images (green + red + DAPI) = 20 µm

Here, the expression of LXRα and LXRβ proteins in the cornea was assessed by immunofluorescence in cornea tissue sections (Fig. 5a, c, d, f) and by Western blot analysis of cornea lysates (Fig. 5b, e). In the native cornea, both LXRα and LXRβ were expressed in the superficial and basal cells of the epithelium and within cells in the stroma. In the sutured cornea, the expression of these receptors was associated with infiltrating inflammatory cells, whereas epithelial expression diminished during sprouting (Fig. 5a, c, d, f). Conversely, during remodeling overall stromal expression decreased, as fewer inflammatory cells were present, while epithelial expression was re-established. With emphasis on the 72-h time point in the remodeling arm, CD45+, CD68+ and CD163+ cells co-expressed LXRα (Fig. 5c) and LXRβ (Fig. 5f). Sub-populations of CD45+ leukocytes expressed both LXRα and LXRβ, including CD68+ monocyte/macrophages and CD163+ remodeling macrophages.

Given the known anti-inflammatory activity of the activated pathways shown in Fig. 4a [19, 20], next was to assay for the expression of known pro-inflammatory genes, potentially downstream of these pathways.

### Genes downstream of LXR and prototypical inflammatory genes are differentially regulated in corneal tissue during remodeling versus sprouting

**Fig. 6:**
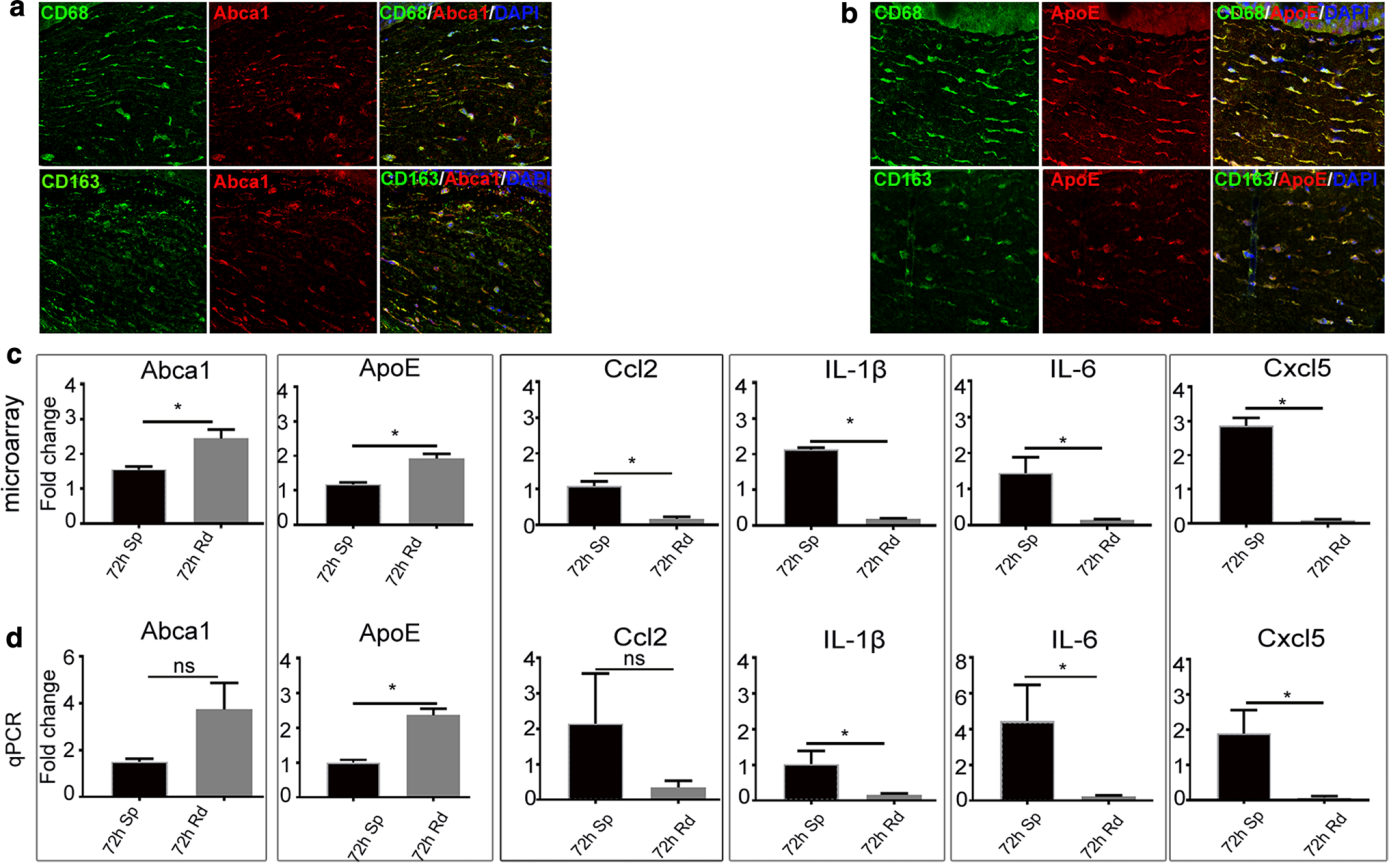
Expression of genes downstream of LXR, Abca1 and ApoE in cornea tissue sections, and fold change expression of downstream pro-inflammatory genes. **a** and **b** are the localization of expression of Abca1 and ApoE, respectively, in cells within cornea sections at 72 h in the remodeling arm. In a, the green color represents CD68 and CD163, while the red color represents Abca1. In **b**, the green color represents CD68 and CD163, while the red color represents ApoE. **c, d** are the expression profiles of LXR target genes, and pro-inflammatory genes assayed by microarray (c) and qPCR (d). In both **c** and **d**, the error bars represent SEM, and ‘ns’ represents no significant difference in the fold change at 72 h between the sprouting and remodeling arms. The asterisk represents *p* < 0.05 between the sprouting arm (abbreviated as 72-h Sp) and remodeling arm (abbreviated as 72-h Rd). For both the sprouting and remodeling arms, *n* ≥ 4 at each time point

The expression of the downstream targets of LXR activity: ATP-binding cassette transporter a1 (Abca1) [21–23] and apolipoprotein E (ApoE) [23] was observed in cornea cross sections in Fig. 6a, b, respectively, associated with CD68+ and CD163+ cells. The expression trend of Abca1 and ApoE observed by microarray was mirrored by qPCR (Fig. 6c, d). Studies investigating LXR and PPAR activation have shown inhibitory effects on pro-inflammatory genes [24–26]. Here, the expression of cytokines that we [16] and others [27, 28] have previously shown to be upregulated in inflammatory corneal angiogenesis was examined. By qPCR, it was observed that C-X-C motif chemokine ligand 5 (Cxcl5), chemokine (C–C motif) ligand 2 (Ccl2), interleukin-6 (IL-6) and interleukin-1 beta (IL-1β) were suppressed in remodeling capillaries compared to the sprouting arm at 72-h time point, confirming the trend in gene expression from the microarray data (Fig. 6c, d).

To gain insight into the regulatory mechanisms driving the observed pathway enrichment, we performed an upstream regulatory analysis in the remodeling arm across time points 24, 72 and 120 h.

### Upstream regulatory analysis of the remodeling response

**Fig. 7:**
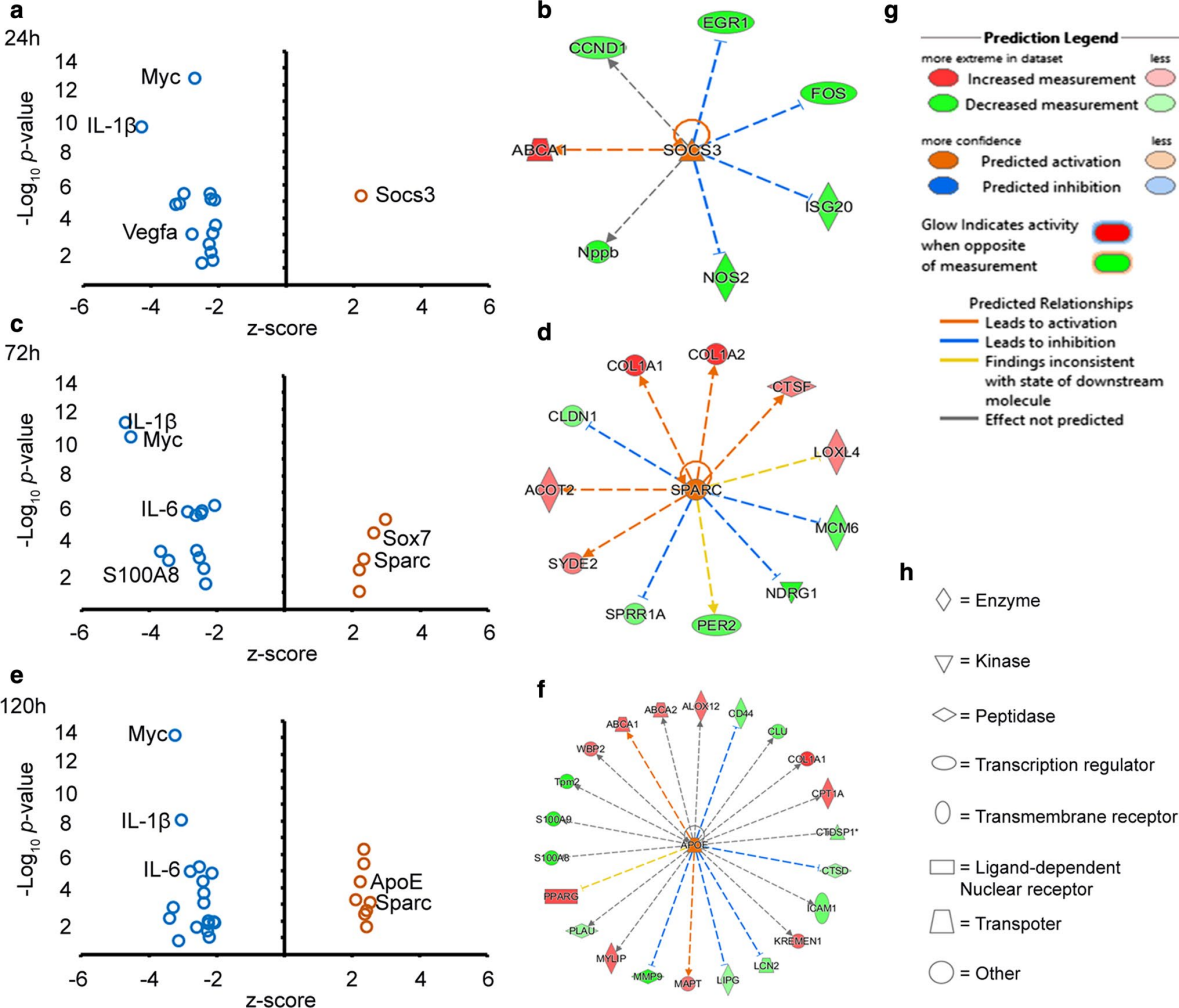
Upstream regulatory analysis of genes and their mechanistic activity in the remodeling arm over time. **a**, **c** and **e** are volcano plots showing modulated upstream regulators, putatively driving the gene expression patterns at 24, 72 and 120 h, respectively. The blue and orange small circles represent inhibited (represented by negative *z*-score values) and activated (represented by positive *z*-score values) upstream regulatory genes, respectively. **b**, **d** and **f** are mechanistic networks for Socs3, Sparc and ApoE, respectively, where the dashed lines represent an indirect interaction. The orange dashed line indicates ‘leads to activation,’ the blue dashed line indicates ‘leads to inhibition,’ the yellow dashed line indicates ‘findings inconsistent with state of downstream molecule,’ and the gray dashed line indicates ‘effect not predicted.’ These predictions are further illustrated in the prediction legend in **g**. In **h** are network shapes describing the type of molecule involved in the mechanistic network. The prediction legend and network shapes were extracted from Ingenuity Pathways Analysis (IPA software, Inc.)

Upstream regulatory analysis is built on predictions of the activity of the encoded protein for a given upstream regulator rather than around the gene expression profile of the molecule (IPA software, Inc.). This analysis helps to identify regulatory molecules driving the observed gene expression profiles in the experimental data. Here, we analyzed for upstream regulators with a bias toward the observed preferential activation of LXR/RXR and PPAR signaling pathways in remodeling. Upstream regulatory analysis at 24 h revealed suppressor of cytokine signaling 3 (Socs3) as the only activated upstream regulator (activation *z*-score 2), with MYC proto-oncogene, bHLH transcription factor (Myc) and IL-1β being the most inhibited regulatory molecules (inhibition *z*-score − 2 and − 4.5, respectively) (Fig. 7a). A mechanistic analysis revealed that Socs3 activates Abca1, one of the central molecules for the regulation of the inflammatory response by LXR’s (Fig. 7b). At 72 h, Myc and IL-1β were further inhibited (inhibition *z*-score − 4.2 and − 4.1, respectively), while the gene secreted protein acidic and cysteine rich (Sparc) among others was activated (activation *z*-score 2) (Fig. 7c). At the mechanistic level, it was found that Sparc activated collagens Col1a1 and Col1a2 among others (Fig. 7d). At 120 h, Myc and IL-1β were inhibited as observed earlier at 72 h. On the other hand, ApoE and Sparc were activated (activation *z*-score 2 and 2.5, respectively) (Fig. 7e). Mechanistically, we found ApoE to regulate many genes, but most importantly, to activate Abca1 and Abca2, genes involved in LXR activity (Fig. 7f).

**Fig. 8:**
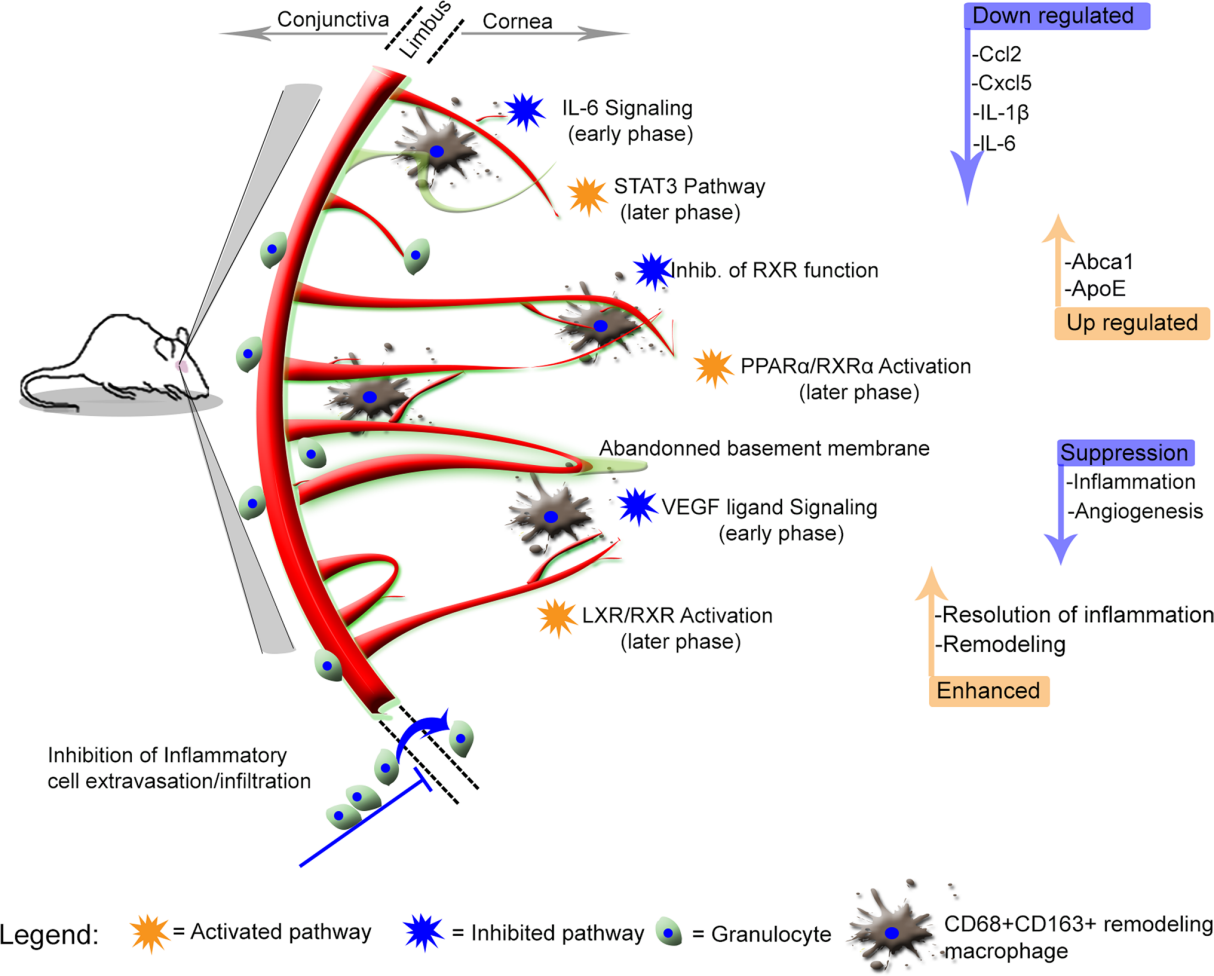
Graphical summary of the time-dependent modulation of induced remodeling of corneal capillaries. Capillary remodeling in the cornea is a time-dependent process involving the early inhibition of pro-inflammatory and pro-angiogenic pathways like VEGF ligand signaling and IL-6 signaling, and in a later phase the activation of LXR/RXR, PPARα/RXRα and STAT3 pathways. This ultimately leads to an inhibition of pro-inflammatory genes such as Ccl2, Cxcl5, IL-1β and IL-6. The suppression of these genes dampens inflammation and angiogenesis, to promote remodeling of capillaries, and the establishment a functional and persistent corneal vascular network. Remodeling-type macrophages promote remodeling through phagocytosis and activation of LXR pathway and the downstream target genes. The blue straight arrows represent downregulation/suppression, while the brown straight arrows represent upregulation/enhanced. The blue curved arrow represents inhibition of cell migration from the conjunctiva into the cornea

In summary, inducing capillary remodeling by removing the angiogenic stimuli leads to an early inhibition of pro-inflammatory and pro-angiogenic pathways (for example VEGF ligand signaling and IL-6 signaling). In a later phase, a subset of pathways are activated (for example LXR/RXR, PPARα/RXRα and STAT3 pathways), which are potentially responsible for the suppression of pro-inflammatory genes such as Cxcl5, Ccl2, IL-6 and IL-1β. During this phase, remodeling macrophages appear in the cornea and presumably clear debris from apoptotic endothelial and immune cells and aid in pruning and remodeling of capillaries [15]. Interestingly, phagocytosis in macrophages is known to activate LXRs and to regulate the expression of downstream targets such as ApoE and Abca1 [29]. Expression of these factors in turn leads to enhanced cholesterol efflux from the cells and also leads to the transrepression [30] of NF-κB signaling [31, 32] to suppress the expression of pro-inflammatory genes [31, 33]. In this study, ApoE and Abca1 expression by CD68+ CD163+ remodeling macrophages provides further evidence of anti-inflammatory properties of these cells, possibly triggered by phagocytosis. Subsequent suppression of downstream pro-inflammatory genes leads to a suppression of inflammation and an increased remodeling of corneal capillaries to establish a functional and persistent vasculature as observed in this study (Fig. 8).

## Discussion

Here we investigated the time dependence of inflammatory pathways involved in angiogenic sprouting and remodeling of corneal capillaries. Capillary remodeling in the cornea initiated by removing the inflammatory and angiogenic stimulus enhanced constriction of corneal capillaries in a time-dependent manner and inhibited infiltration of inflammatory cells as shown by IVCM. Time dependence at the tissue level was also reflected at the transcriptome level, where remodeling was characterized by stronger and more widespread suppression of genes with time. In the remodeling arm, as expected an immediate (24 h) inhibition of pro-inflammatory and pro-angiogenic pathways (such as IL-6, IL-8, CXCR4, ILK, VEGF ligand-receptor signaling and endothelin signaling) was observed following removal of the stimuli. IL-8 (CXCL8) signaling, important for neutrophil activation [34], and secreted by many cells including endothelial cells [35] was still active at both 72- and 120-h time points in the sprouting arm. ILK signaling, another pro-inflammatory pathway [36], was active in the sprouting arm from 72 to 120 h, while the pathway was inhibited early at 24 h in the remodeling arm. Signaling through ILK is implicated in immune cell trafficking and survival [37], processes that are important for sustained inflammation and angiogenesis. VEGF ligand-receptor signaling is a well-described pathway that modulates the effects of VEGFA [38] in angiogenesis, and inhibition of this pathway at the earliest time point in the remodeling arm could indicate that remodeling of corneal capillaries is VEGF-independent, or that resolution of inflammation and remodeling processes can proceed only after VEGF signaling is suppressed. Pathway analysis indicated shutting down of other pro-angiogenic and pro-inflammatory pathways was also effected before the onset of remodeling at the tissue level. By 72 h after initiation of remodeling—when remodeling at the tissue level became apparent—a 50:50 inhibition/activation of pathways was observed that signified a new ‘phase’ in the remodeling process.

A main finding in this study was that from 72 h and onward a synergistic relationship was observed between ‘LPS/IL-1 inhibition of RXR function’ (inhibited) and LXR/RXR signaling (activated). This finding illustrates the interplay between two opposing pathways in regulating inflammation and likely the remodeling of capillaries. Moreover, LXR/RXR activation and peroxisome proliferator-activated receptor-α (PPARα)/RXRα pathways were found to be active during remodeling at 72 and 120 h, but both were inhibited in the sprouting arm at 72- and 120-h time points. LXR/RXR activity is reported to have anti-angiogenic [39] and anti-inflammatory [24] effects. Expression of LXR in endothelial cells is known [40], and LXRβ has been shown to prevent endothelial cell senescence [41]. In relation to this study, expression of LXR by remodeling vessels could be a mechanism to promote their persistence. Retinoid X receptors (RXRs) partner with liver X receptor (LXR) to modulate the transcription of many genes, and there is mounting evidence to suggest the role of LXRs in innate and adaptive immunity and inflammation [42].

Here, LXRα was expressed weakly in early infiltrating CD45+ leukocyte granulocytes, but a stronger expression was observed in CD68+ CD163+ remodeling macrophages in the remodeling arm. LXRβ was similarly expressed weakly by early infiltrating CD45+ leukocyte granulocytes. Furthermore, LXRβ was expressed in some CD68+ CD163+ remodeling macrophages in the remodeling arm. In line with these findings, previous studies have shown that expression and activation of LXRs in human lymphocytes reduces pro-inflammatory signaling [43], and activation of LXR using synthetic agonists in monocytes promotes anti-inflammatory properties [44]. Monocyte transition to macrophages and LXR activation have been shown to polarize macrophages to the M2 phenotype [45]. Stimulation of macrophages by either TNF-α, LPS or IL-1β represses inflammatory genes such as Ccl2, Ccl7 and Mmp9 [24, 32], a response mediated by LXRs. In another study, activation of LXRα attenuated ocular inflammation through the inhibition of NF-κB signaling pathway [46]. Furthermore, activation of LXR suppresses angiogenesis through induction of ApoD [47]. Activation of LXR leads to an increased expression of Abca1 [22] and Abcg1, proteins important for cholesterol efflux from cells [48]. In relation to inflammation, Ito et al. showed that LXR inhibits NF-kB and MAPK signaling by disrupting membrane lipid organization through Abca1. Abca1 is important for the activation of JAK2, which in turn activates STAT3 [49]. In the present study, a time-dependent expression of Abca1 was apparent in the remodeling arm. The expression of Abca1 was significantly different between the sprouting and remodeling arms at 72 h as shown by microarray analysis. In cornea cross sections at 72 h within the remodeling arm, CD68+ CD163+ remodeling macrophages were shown to express Abca1. The expression of Abca1 in these cell types can be linked with promoting anti-inflammatory signaling, based on knowledge that Abca1 is a target gene for LXRs to enhance cholesterol efflux and to promote anti-inflammatory properties in macrophages [21]. Studies in murine macrophages documented the interruption of (NF)-kb signaling by LXR by transrepression [24]. LXR agonists like GW3965 and TO901317 are shown to interfere with the expression of inflammatory genes in dendritic cells [50].

Signal transducers and activators of transcription 3 (STAT3) were another pathway of interest, activated in the remodeling arm earlier than in the sprouting arm. In the eye, STAT3 is important for the development of the retina [51] and is also associated with retinal neovascularization [52]. Activation of STAT3 and cholesterol efflux from macrophages has been shown to contribute to anti-inflammatory properties [53]. Furthermore, it is thought that a combination of cholesterol efflux and activation of STAT3 is key for the anti-inflammatory properties of the Abca1/apoA-1 axis [53]. PPARα, another activated pathway in remodeling, is one of the peroxisome proliferator-activated receptors (PPARs) and dimerizes with RXR [54]. PPARs are anti-proliferative and anti-angiogenic [55]. Clinically, PPARα agonists are used to inhibit proliferation and angiogenesis [56]. Wy-14643, a PPARα agonist, was shown to reduce tumor vascularization and growth through the inhibition of endothelial cell proliferation in mice [57]. PPARγ is reported to repress monocyte transmigration and macrophage inflammatory response [58]. Among other activators, PPARγ can be activated by a laminar flow which in turn upregulates LXR in vascular endothelial cells [59]. Furthermore, pioglitazone, a PPARγ agonist, was reported to suppress angiogenesis in the rat cornea [60]. Activation of PPARα/RXRα in the remodeling arm therefore warrants closer attention for its potential role in modulating corneal inflammation.

ApoE, another target gene for LXR [23], was identified as an upstream regulator in this study, whose expression was upregulated in the remodeling arm. ApoE was expressed by CD68+ CD163+ remodeling macrophages at 72 h in the remodeling arm. In line with this finding, it is known that ApoE promotes macrophage polarization toward an anti-inflammatory phenotype by binding to ApoER2 and VLDLR [61]. Our earlier observation of the accumulation of macrophages in the cornea with time during remodeling [15], [16] is a finding that may be attributable to an upregulation of ApoE. As a therapeutic target, ApoE peptides are shown to have anti-inflammatory properties in the cornea [62]. Tang et al. [53] showed that an interaction of ApoA-I/ABCA1 activates cholesterol efflux, and STAT3 branch pathways, to synergistically suppress inflammation in macrophages. Besides the anti-inflammatory properties of ApoE, this protein is also reported to potentially influence angiogenesis [63]. A signaling cascade involving PPARγ-LXR and ApoE is described in other tissues [64], and in line with this, here we observed a mechanistic activation of PPARγ by ApoE, pointing toward a potential anti-inflammatory role.

From the upstream regulatory analysis in the remodeling arm, Socs3 was activated early at 24 h. SOCS are intracellular cytokine-inducible proteins that interfere with cytokine signaling through JAK proteins and/or cytokine receptors or by inhibition of STAT [65]. SOCS block the inflammatory response by mediating the degradation of target proteins [66, 65]. In particular, Socs3 is induced and degraded rapidly and is known to block the activation of STAT3 in response to IL-6, by binding to the IL-6 gp130 receptor complex and mediating its degradation [67, 68]. Furthermore, it has been shown that Socs3 attenuates pro-inflammatory signaling to suppress acute inflammation [69]. It is thought that high Socs3 expression is associated with M1 pro-angiogenic macrophages, and in line with this, we previously showed an increased presence of inflammatory cells (monocyte/granulocytes) at 24 h in inflammatory corneal angiogenesis [16]. In the present study, the activation of Socs3 coincided with a start in the reduction of inflammation, thus highlighting a potential anti-inflammatory role of Socs3 in this model. Statin-induced Socs3 expression is shown to downregulate IL-1*β* [67], a result in agreement with the observed downregulation of IL-1*β* in this study. Mechanistically, we found that Socs3 activates Abca1, an observation that is corroborated by studies which show that the anti-inflammatory effect of the apoA-I/ABCA1/STAT3 pathway is Socs3 dependent [53]. In relation to the observed pathway enrichment, Xiong et al. [70] showed that the activation of LXR induced the expression of Socs3, and to illustrate a potential dual anti-inflammatory and anti-angiogenic property of Socs3, Stahl et al. [71] showed Socs3 to have inhibitory effects on pathologic angiogenesis in murine models of oxygen-induced retinopathy and cancer. At the pathway level, activation of LXR is reported to induce the expression of Socs3 to inhibit cell proliferation, a response specific to LXRα-SOCS3-cyclin D1/p21/p27 signaling pathway [70].

Secreted protein acidic and rich in cysteine (Sparc) is another upstream regulator and was activated at both 72 and 120 h during remodeling. Sparc is known to regulate inflammation and collagen deposition [72], and the absence of Sparc is associated with an increased inflammatory cell infiltration [73] and a reduction in regulating cytokine production [74]. These reports provide a potential explanation for the observed mechanistic interaction between Sparc and collagens (Col1A1, Col1A2) as observed here. Furthermore, activation of Sparc could also be responsible for the reduction in the overall inflammatory cell infiltration as observed by IVCM in the remodeling arm in this study. In a report by Lane et al. [75], addition of synthetic SPARC to endothelial cells resulted in decreased expression of fibronectin and thrombospondin-1, and an increase in the type-1 plasminogen activator inhibitor, hence regulating the different components of the extracellular matrix (ECM). SPARC is also reported to regulate endothelial cell shape and barrier function to facilitate the extravasation of macromolecules [76]. It is however important to keep in mind that the exact role of Sparc could be tissue- and source-dependent.

It is important to investigate whether the pathways activated in this study act together or independently, in order to gain a better understanding of capillary remodeling in the cornea. In the eye, diseases such as AMD are linked to genes involved in metabolism regulated by LXRs [77], and T0901317 (an LXR agonist) is reported to ameliorate retinal inflammation [78]. Our study therefore expands knowledge of inflammatory pathways beyond the retina [77], providing insights into the mechanisms regulating persistent corneal capillaries and motivation for the use of LXR or PPAR agonists for treating corneal inflammatory angiogenesis. In support of this, agonists with broader clinical indications are under investigation, and recently a patent that covers corneal arcus among other indications of an LXR agonist was filed [79]. However, the adverse side effects associated with LXR agonists are a major drawback for clinical use, and this issue needs to be addressed in future research. Furthermore, limiting the effects of LXR agonists to inflammation alone could be a major challenge, given that these receptors are involved in the regulation of other important biological processes as well. To limit the adverse side effects of LXR agonists, strategies such as site-specific antibody drug conjugates have been tested to selectively deliver LXR agonists to their targets, with minimal side effects [80]. For corneal use, the development of a topically applied formulation given as eye drops could minimize exposure to other tissues and limit side effects.

## Conclusion

In this study, we found that resolution of inflammation in the cornea is a time-dependent process, characterized by disappearance of inflammatory cells from the stroma, thinning of the neovessels and a strong downregulation of pro-angiogenic and pro-inflammatory pathways and suppression of inflammatory genes such as Cxcl5, IL-1β, IL-6 and Ccl2. A progressive activation of LXR/RXR, PPARα/RXRα and STAT3 pathways following the initial suppression of VEGF signaling and other angiogenic and inflammatory pathways could be responsible for the resolution of inflammation and the capillary remodeling observed in this study. Investigation of these pathways and their interactions deserves closer attention. Factors such as Socs3, Sparc and ApoE may be upstream regulators of these processes and also warrant further investigation.

## Materials and methods

### Animals and procedures

The Regional Ethics Committee for Animal Experiments at Linköping University, Sweden, issued ethical permission for the animal experiments (permit nos. 7-13 and 585), and all experimental procedures adhered to the guidelines of the Association for Research in Vision and Ophthalmology (ARVO), for the Use of Animals in Ophthalmic and Vision Research. Wistar rats 5–6 weeks old (Scanbur AB, Sollentuna, Sweden) were quarantined and housed at the Center for Biomedical Resources, Linköping University. A standard dark–light cycle of 12:12 h was used. Prior to surgical procedures, general anesthesia was given using a combination of Ketanest (ketamine 25 mg/ml, Pfizer) and Dexdomitor (dexmedetomidine hydrochloride 0.5 mg/ml, Orion Pharma). To induce inflammatory corneal angiogenesis, two nylon 10-0 sutures were placed intrastromally on the temporal side of the right eye cornea and were maintained over four days. On day four (time point 0 h), rats were split into a sprouting arm (where sutures were left in place to provide a sustained stimulus) or a remodeling arm (where both sutures were removed to induce capillary remodeling). Sprouting and remodeling arms were then further examined longitudinally at 24, 72 and 120 h.

### In vivo confocal microscopy

In vivo confocal microscopy (IVCM) is widely used to monitor cellular infiltration into the cornea, with early inflammatory cells characterized as hyper-reflective-rounded or spindle-like structures in the stroma [3], while mature macrophages appear as large polymorphic cells [81]. Here, IVCM was used for longitudinal live imaging of capillary perfusion and cellular infiltration into the corneal stroma. Of the acquired IVCM image sequences, three representative images per biological sample and three biological sample per time point were used to measure the diameter of the capillaries using ImageJ (National Institutes of Health, Bethesda, USA http://rsb.info.nih.gov/ij/index.html), using a method described elsewhere [82]. The results were analyzed using Graph Prism 7 for Windows (GraphPad software, La Jolla California USA, www.graphpad.com).

### Microarray target preparation and hybridization

Four biological samples were used at each time point, and each biological sample corresponded to a single microarray chip, i.e., no pooling of biological samples. Total RNA was extracted from corneal lysates using RNeasy Mini Kit (Qiagen, Hilden, Germany). The RNA was quantified by NanoDrop 2000 (Thermo Scientific) and quality verified using an Agilent 2100 Bioanalyzer (Agilent Technologies Inc., Paolo Alto, CA, USA). RIN value of ≥ 7 was the cutoff for sample inclusion for microarray processing. Single-stranded cDNA targets for microarray hybridization were prepared according to the manufacturer’s protocol (GeneChip^®^ WT PLUS Reagent Kit, P/N 703174 Rev. 2, Affymetrix Inc.). The prepared single-stranded cDNA was hybridized to GeneChip Gene 2.0 ST 100-Format Array (Affymetrix Inc.) in a hybridization oven, washed and scanned.

### Hierarchical cluster analysis and differentially expressed genes

The raw collections of expression array feature intensity (CEL) files for 0 h and for 24 h in the sprouting and remodeling arms were retrieved from NCBI Gene Expression Omnibus (GSE81418). All the CEL files were normalized by the RAM method using Affymetrix Expression Console (Affymetrix Inc.). Hierarchical cluster analysis was performed using chp files, using the Transcriptome Analysis Console (Affymetrix Inc.), with ANOVA *p* ≤ 0.05. To define deferentially expressed genes (DEGs), 0 h was used as the baseline, since 0-h time point is when both inflammation and angiogenesis are high in this model, to isolate mechanisms behind the time-dependent modulation of inflammation and angiogenesis. Relative to 0 h, DEGs at 24, 72 and 120 h were obtained using filters fold change (FC) ≥ 1.5 or ≤ −1.5 and *p* value < 0.05.

### Canonical pathway enrichment analysis

Using the obtained DEGs as the input files, QIAGEN’s Ingenuity^®^ Pathway Analysis (IPA) (IPA^®^, QIAGEN Redwood City, CA) software was used for canonical pathway and upstream regulatory analysis. The core analysis was performed using default parameters to map the DEGs to their corresponding objects in the Ingenuity Pathways Knowledge Database, to build biological relationships among the DEGs. Following the core analysis, canonical pathway analysis was performed to identify activated/inhibited pathways. The resultant canonical pathways were compared between the sprouting and remodeling arms longitudinally. Upstream regulatory analysis was performed in the remodeling arm to identify potential targets responsible for the observed canonical pathway enrichment.

### Immunofluorescence staining

Following fixation of corneal tissue in 4% PFA, the tissue was embedded in paraffin in preparation for sectioning. Five-micrometer-thick paraffin sections were made from the paraffin blocks, mounted on to a slide and deparaffinized in xylene and rehydrated in decreasing alcohol concentrations. Antigens were retrieved in heated citrate buffer (10 mM, pH 6) for 5 min. The samples were permeabilized with cold acetone for 20 min at − 20 °C and treated with 0.1% Triton-×100 in PBST for 10 min at RT. Signal enhancer was used to pre-block samples for 30 min at RT, prior to blocking with 1% BSA in PBST for 2 h at RT. Primary antibodies against LXRα (1:500, Abcam: ab3585), CD45 (1:10 Abcam: ab86080), CD68 (1:50 GeneTex: GTX41868), CD163 (1:500 Abcam: ab182422), ApoE (1:500 Abcam: ab20874), Abca1(1:500 Abcam: ab18180) and LXRβ (1:500 Abcam: ab28479) were applied overnight at 4 °C in a humidified chamber. Fluorescently labeled secondary antibodies (Alexa 488, Thermo Fisher Scientific, MA, USA, and Alexa 594) diluted 1:1000 were applied for 1 h at RT. For double staining, the primary antibody (for the first target) was probed using a fluorescently labeled secondary antibody at RT for 2 h, washed and incubated again overnight at 4 °C with another primary antibody (for the second target). The next day, slide was washed in the dark and probed with another (with fluorochrome different from the first) fluorescently labeled secondary antibody at RT for 1 h. Slides were washed and mounted with ProLong Gold antifade reagent with DAPI (Invitrogen, Thermo Fisher Scientific, MA, USA). Images were captured using LSM 700 laser confocal microscope (Carl Zeiss).

### Real-time PCR analysis

Total RNA was extracted as described above. Following cDNA synthesis (Superscript III VILO cDNA synthesis kit: Invitrogen Life Technology, MA, USA), quantitative PCR was performed using SYBR Green (Applied Biosystems, CA, USA) chemistry, with primers for Abca1, ApoE [83] and Ccl2 [84] (Supplementary table I). For IL-1β, IL-6 and Cxcl5 (PrimeTime, Integrated DNA Technologies), custom-designed primer sequences were used with the TaqMan Advanced Master Mix (Applied Biosystems, CA, USA). Threshold cycle Ct values were normalized to Gapdh, and gene fold change was determined by the relative comparison method, relative to the 0-h time point.

### Western blot analysis

Cornea tissue in RIPA buffer supplemented with 1% protease inhibitors (Roche Diagnostics) was lysed using a tissue disruptor with metal beads (Qiagen, Hilden, Germany). Lysates were prepared in RIPA buffer, and 18 ug of total protein was separated on 4–20% Mini Precast Gels (Bio-Rad, CA, USA). Semi-dry transfer using trans-blot turbo system (Bio-Rad, CA, USA) with pre-set mixed-MW settings was used to transfer proteins onto a PVDF membrane. The membranes were blocked in 5% non-fat milk for 1 h at RT. Membranes were probed with antibodies against LXRα (1:300, Abcam: ab3585) and LXRβ (1:500, Abcam: ab28479) O/N at 4 °C. Specific HRP-conjugated secondary antibody was used (1:1000) (AP307P, 2700944, AP308P, 2688593; 1:1000; Merck Millipore, MA, USA) and detected by chemiluminescence (Bio-Rad, CA, USA). The signals were captured with an ImageQuant LAS 500 gel imaging system (General Electric, CT, USA).

### Statistical analysis

Analysis of variance (ANOVA) with Dunn’s multiple comparison tests was used to compare more than two-sample means. The unpaired Student t test was used whenever comparing two-sample means. A *p* value < 0.05 was considered significant in both ANOVA and t test. The data are presented as the mean, with error bars representing a standard error of the mean (SEM). The microarray data were sorted on *p* value < 0.05 to filter for DEG, and ANOVA was used for multiple comparison.

## Electronic supplementary material

Below is the link to the electronic supplementary material.

Supplementary material 1 (DOCX 2372 kb)
