# Supplementary material for: Time-dependent LXR/RXR pathway modulation characterizes capillary remodeling in inflammatory corneal neovascularization
Source: Angiogenesis. 2018 Feb 14;21(2):395–413. doi: 10.1007/s10456-018-9604-y (PMC5878196; doi:10.1007/s10456-018-9604-y)
Supplement: Supplementary file 1 — Supplementary material 1 (DOCX 2372 kb) [file 10456_2018_9604_MOESM1_ESM.docx]

**Supplementary Material**

**Time-dependent LXR/RXR modulation characterizes capillary remodeling in inflammatory corneal neovascularization**

Anthony Mukwaya^1^ Anton Lennikov^1^, Maria Xeroudaki ^1^, Pierfrancesco Mirabelli^1^, Mieszko Lachota^2^, Lasse Jensen^3^, Beatrice Peebo^1^ and Neil Lagali^1*^

^1^Department of Ophthalmology, Institute for Clinical and Experimental Medicine, Faculty of Health Sciences, Linkoping University, 58183 Linköping, Sweden

^2^Department of Immunology, Medical University of Warsaw, Warsaw, Poland

^3^Department of Medical and Health Sciences, Division of Cardiovascular Medicine, Linköping University, Linköping, Sweden

*Corresponding author:

Neil Lagali, PhD

Department of Ophthalmology

Institute for Clinical and Experimental Medicine

Faculty of Health Sciences

Linkoping University,

58183 Linköping, Sweden

Tel +46 101034658

Fax +46 101033065

[neil.lagali@liu.se](mailto:neil.lagali@liu.se)

Supplementary Fig.1. IVCM image sequences illustrating time dependence of infiltrating inflammatory cells, perfusion of neovessels, and the presence of macrophages.

**
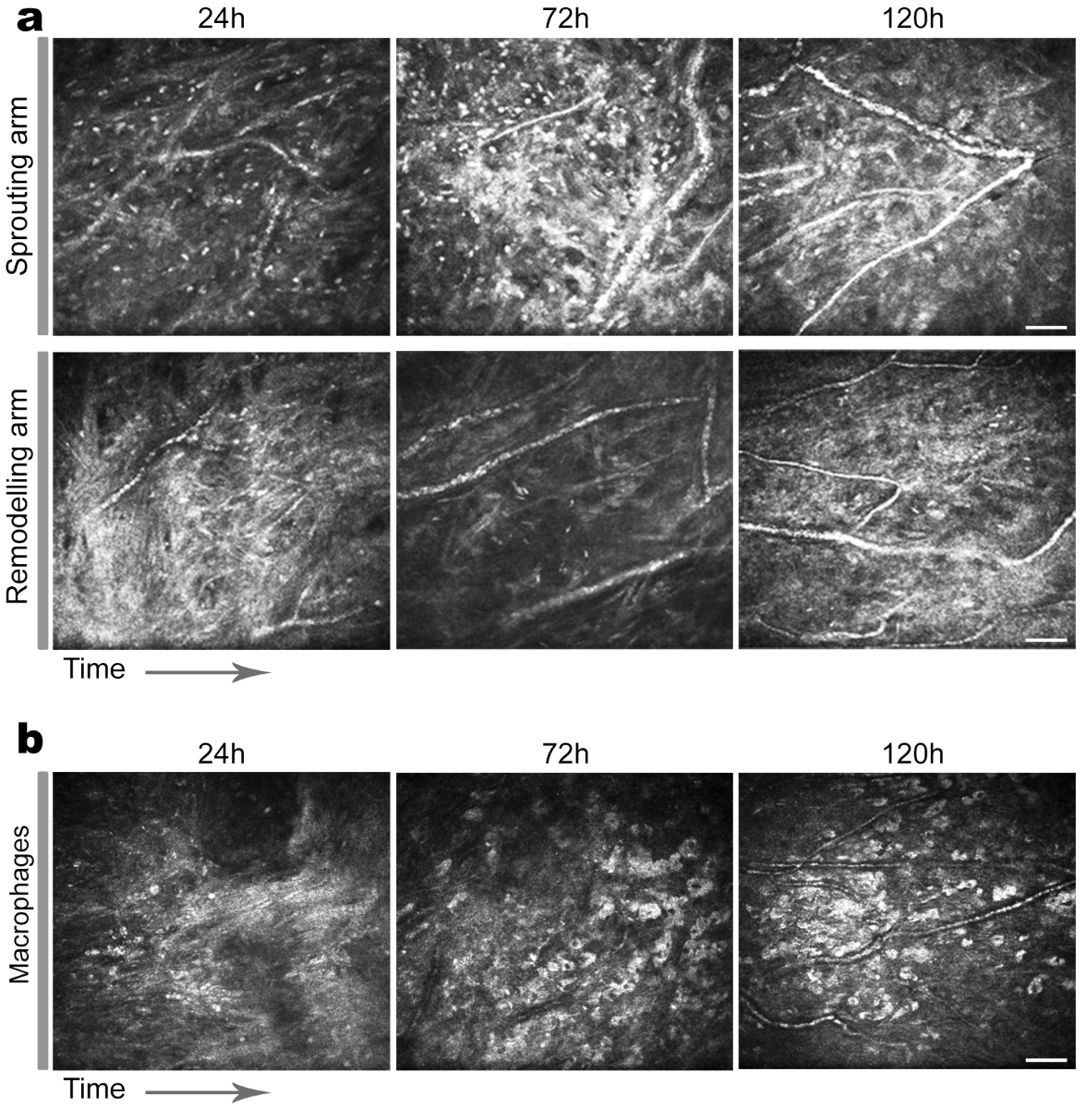
**

**Supplementary Fig.1.** Representative *In vivo* confocal microscopy image sequences illustrating inflammatory cell infiltration into the cornea, and the variation in diameter of neovessels. **a** illustrates early inflammatory cell infiltration (predominantly granulocytes), and perfused neovessels in the sprouting and remodeling arms with time. **b** illustrates a time dependent variation in macrophages in the remodeling arm. In both **a** and **b**, the scale bar= 50µm.

Supplementary Fig.2. Hierarchical cluster analysis and the comparison of the DEGs between the corresponding time points in the sprouting and remodeling arm.


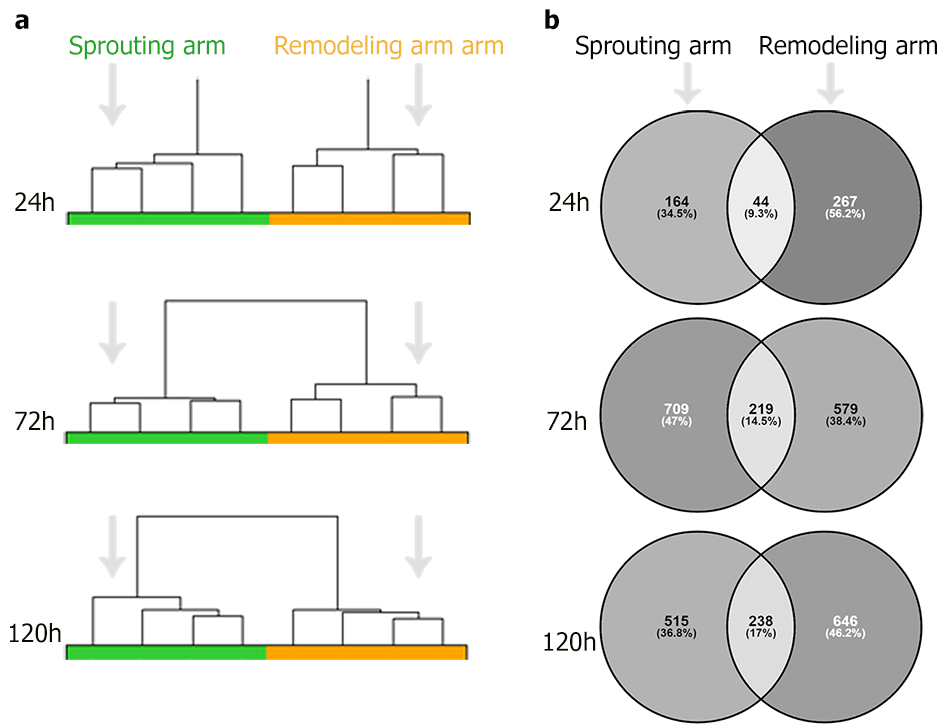


**Supplementary Fig.2.** **a** Hierarchical cluster analysis differentiating between the sprouting and remodeling arms across time points. The hierarchical clusters from the analysis of the samples using the CHP files generated from normalising the microarray raw CEL files **b** Comparison of the number of DEGs across time points in the sprouting and remodeling arms.


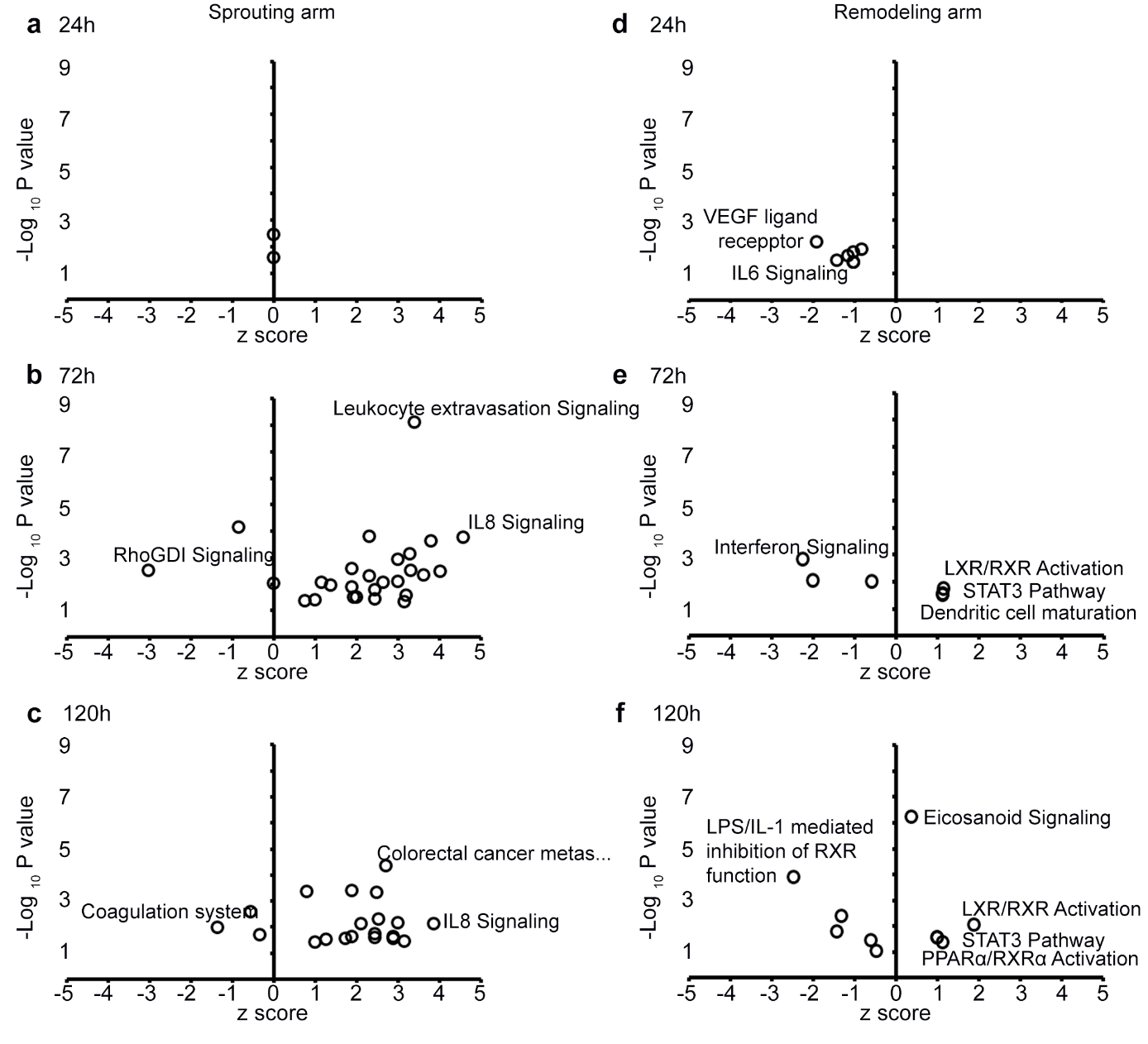


**Supplementary Fig.3.** Canonical pathway modulation across time points. **a-c** are the canonical pathways modulated in the sprouting arm. **d-f** are the canonical pathways modulated in the remodeling arm. On the x-axis is the activity z-score, with the negative z-score values representing the inhibited pathways while positive z-score values representing activated pathways. On the y-axis is the significance values with the –log (p-value) displayed along the y-axis, with a p ˂ 0.05 as the cut-off. The greater the –log (p-value), the more significant the overlap between the DEGs and the pathway

**Supplementary Table I.** Primer Sequences used for SYBR Green qPCR analysis.

| **Gene Symbol** | **Forward** | **Reverse** |
| --- | --- | --- |
| Abca1 | CTCGAATTATTTGGAAGGCAC | TTTGGGGACTGAACATCCTCT |
| ApoE | GGAACTGACGG TACTGATGGA | TCGGATGCGG TCACTCAAA |
| Ccl2 | AGGCAGATGCAGTTAATGCCC | ACACCTGCTGCTGGTGATTCTC |
